# Study of *Plasmodium falciparum* DHHC palmitoyl transferases identifies a role for PfDHHC9 in gametocytogenesis

**DOI:** 10.1111/cmi.12599

**Published:** 2016-05-03

**Authors:** Chwen L. Tay, Matthew L. Jones, Nicola Hodson, Michel Theron, Jyoti S. Choudhary, Julian C. Rayner

**Affiliations:** ^1^ Malaria ProgrammeWellcome Trust Sanger InstituteCambridgeUK; ^2^ Proteomic Mass SpectrometryThe Wellcome Trust Sanger InstituteThe Wellcome Trust Genome CampusHinxtonCambridgeCB10 1SAUK

## Abstract

Palmitoylation is the post‐translational reversible addition of the acyl moiety, palmitate, to cysteine residues of proteins and is involved in regulating protein trafficking, localization, stability and function. The Aspartate‐Histidine‐Histidine‐Cysteine (DHHC) protein family, named for their highly conserved DHHC signature motif, is thought to be responsible for catalysing protein palmitoylation. Palmitoylation is widespread in all eukaryotes, including the malaria parasite, *Plasmodium falciparum*, where over 400 palmitoylated proteins are present in the asexual intraerythrocytic schizont stage parasites, including proteins involved in key aspects of parasite maturation and development. The *P. falciparum* genome includes 12 proteins containing the conserved DHHC motif. In this study, we adapted a palmitoyl‐transferase activity assay for use with *P. falciparum* proteins and demonstrated for the first time that *P. falciparum* DHHC proteins are responsible for the palmitoylation of *P. falciparum* substrates. This assay also reveals that multiple DHHCs are capable of palmitoylating the same substrate, indicating functional redundancy at least *in vitro*. To test whether functional redundancy also exists *in vivo*, we investigated the endogenous localization and essentiality of a subset of schizont‐expressed PfDHHC proteins. Individual PfDHHC proteins localized to distinct organelles, including parasite‐specific organelles such as the rhoptries and inner membrane complex. Knock‐out studies identified individual DHHCs that may be essential for blood‐stage growth and others that were functionally redundant in the blood stages but may have functions in other stages of parasite development. Supporting this hypothesis, disruption of PfDHHC9 had no effect on blood‐stage growth but reduced the formation of gametocytes, suggesting that this protein could be exploited as a transmission‐blocking target. The localization and stage‐specific expression of the DHHC proteins may be important for regulating their substrate specificity and thus may provide a path for inhibitor development.

## Introduction

*Plasmodium falciparum* is the most virulent of the malaria‐causing *Plasmodium* parasites, causing the majority of malaria‐associated deaths (Greenwood *et al.,* 2008; Winzeler, 2008). The life cycle of *P. falciparum* is complex, involving development in both a mosquito vector and a human host. However, all symptoms of malaria occur as a result of the intraerythrocytic stages of the parasite life cycle, during which the parasite undergoes asexual replication within human erythrocytes. Intraerythrocytic development and replication are tightly regulated, in part by controlled waves of transcription (Bozdech *et al.,* 2003). While transcriptional regulation can control which proteins are present, development in eukaryotic cells is also frequently modulated by protein post‐translational modifications (PTMs), which can regulate protein activity and function. Several large datasets suggest an important role for phosphorylation during intraerythrocytic stages (Solyakov *et al.,* 2011; Treeck *et al.,* 2011; Lasonder *et al*., 2012a,b; Pease *et al.,* 2013; Collins *et al.,* 2014), but the role of other PTMs in *P. falciparum* development remains largely unexplored (Doerig *et al.,* 2015).

Protein palmitoylation, the reversible addition of palmitate (a 16‐carbon‐saturated fatty acid) to cysteine residues via a thioester linkage, can affect protein trafficking, localization, interaction and stability (Resh, 2006; Linder and Deschenes, 2007). Unlike some other acyl protein modifications, such as N‐myristoylation and prenylation, protein palmitoylation is a reversible process, so it can be used to dynamically regulate protein function (Resh, 2006). There is no consensus sequence for palmitoylation (Smotrys and Linder, 2004), and advances in proteome‐based technologies for the purification and identification of all palmitoylated proteins in an organism have revealed that up to 10% of the proteome is palmitoylated at any one point in time (Yang *et al.* *,* 2010; Roth *et al.,* 2006; Kang *et al.,* 2008; Martin and Cravatt, 2009; Wilson *et al.,* 2010).

The wide range of proteins that can be palmitoylated, coupled with the reversible nature of the thioester bond, suggests that palmitoylation is an essential tool in regulating protein activity and normal cellular function. In yeast, the global protein palmitoylation pattern is altered during meiosis, (Zhang *et al.,* 2013), while in cardiac muscle, the massive endocytosis that occurs during reoxygenation of cardiac tissue following acute ischemic events depends on modulating membrane protein palmitoylation (Hilgemann *et al.,* 2013; Lin *et al.,* 2013). The role of palmitoylation in regulating cellular processes in *Plasmodium* is largely unknown, although a study of *P. falciparum* schizont stages revealed more than 400 palmitoylated proteins (Jones *et al.,* 2012a), including proteins involved in parasite‐specific processes, such as host cell invasion, drug resistance and cytoadherance (Jones *et al*., 2012a,b).

Palmitoylation is catalysed by the Aspartate‐Histidine‐Histidine‐Cysteine (DHHC)‐palmitoyl‐transferases (PATs), all of which contain multiple transmembrane (TM) domains that flank a highly conserved DHHC motif, thought to be the catalytic site (Politis, 2005; Mitchell *et al.,* 2006). Most eukaryotic organisms possess a repertoire of DHHC‐PATs, with 7 DHHC‐PATs in yeast, 23 DHHC‐PATs in humans, 12 in *P. falciparum* and 11 in *Plasmodium berghei* (Fukata *et al.,* 2006; Roth *et al.,* 2006; Jones *et al.,* 2012b). A recent study of the repertoire of DHHC proteins in *P. berghei* and the related Apicomplexan species, *Toxoplasma gondii*, established that individual DHHC proteins are found in distinct organelles, both elements of the secretory pathway such as the endoplasmic reticulum (ER) and the Golgi, as well as parasite‐specific organelles, such as the rhoptries and the inner membrane complex (IMC) (Frénal *et al.,* 2013). Localization of individual DHHC‐PATs to different compartments has also been observed in *P. falciparum* (Wetzel *et al.,* 2015).

Despite these studies of DHHC function and location in *Plasmodium* parasites, it has never been definitively shown that any *Plasmodium* DHHC orthologues have palmitoyl‐transferase activity. In this manuscript, we report the use of biochemical and genetic tools to characterize four *P. falciparum* homologues in order to address key questions in their potential as drug targets – whether they actually contain the predicted enzymatic activity and whether any display biologically significant functions.

## Results

### Design of a *Plasmodium falciparum* palmitoyl‐transferase activity assay

**Figure 1:**
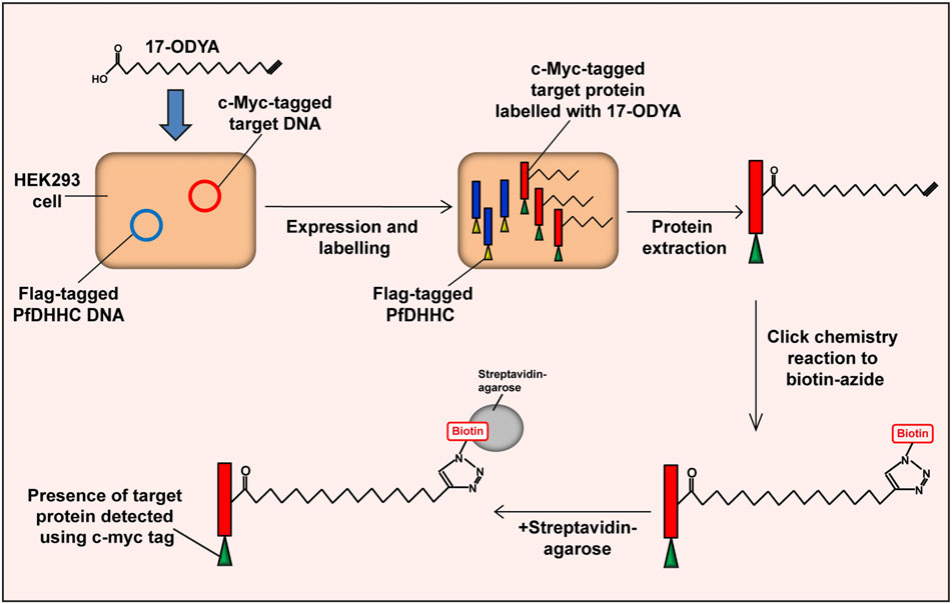
Palmitoyl‐transferase activity assay. This assay incorporates the expression of *Plasmodium falciparum* proteins in a mammalian cell expression system along with metabolic labelling and click chemistry methods of palmitoyl‐protein purification. Human embryonic kidney 293E (HEK293E) cells were co‐transfected with FLAG‐tagged PfDHHC DNA along with the c‐Myc‐tagged DNA of a potential target palmitoyl protein, both of which were codon‐optimized for expression in human cells. The HEK293E cells were then treated with the metabolic label, 17‐octadecynoic acid (17‐ODYA). Proteins were extracted and reacted to biotin‐azide under standard click chemistry conditions, resulting in the biotinylation of all 17‐ODYA‐labelled proteins. Biotinylated proteins were affinity purified using streptavidin‐agarose and the presence of the target protein detected using antibodies against the c‐Myc tag. As a standard control, transfected cells were also mock labelled with DMSO instead of 17‐ODYA. As a control for the background palmitoylation of target proteins by endogenous HEK293E DHHC proteins, cells were also co‐transfected with the c‐Myc‐tagged target protein and the CD4‐expressing control vector, in the absence of the PfDHHC. The level of target protein palmitoylation when in the presence of the PfDHHC compared with when in the absence of the PfDHHC was expected to be an indication of whether the particular PfDHHC was responsible for the palmitoylation of the target protein.

Of the 12 potential *P. falciparum* DHHCs, four ‐PfDHHC3 (PF3D7_1121000), PfDHHC5 (PF3D7_1322500), PfDHHC7 (PF3D7_0528400) and PfDHHC9 (PF3D7_1115900)‐ have transcriptional profiles with peak expression in the schizont stages (Le Roch, 2003). To test whether these four proteins actually had palmitoyl‐transferase (PAT) activity, we adapted and expanded a PAT activity assay developed for other eukaryotic PATs (Hicks *et al.,* 2011). As outlined in detail in Fig. 1, the assay relies on co‐expression of epitope‐tagged PfDHHC and target proteins in HEK293E cells, and a combination of immunoprecipitation and metabolic labelling using the palmitic acid analogue, 17‐octadecynoic acid (17‐ODYA). The 17‐ODYA‐labelled proteins can then be biotinylated via click chemistry, allowing their purification with streptavidin‐agarose (Martin and Cravatt, 2009), and the detection of *Plasmodium* targets within this palmitoylome via the epitope tag. Two potential target proteins were used in combination with all four PfDHHCs; PfSec22 (PF3D7_0320100), a TM‐domain‐containing SNARE protein localized predominantly to the ER (Ayong *et al.,* 2009), which we have detected in palmitoylome datasets, and PfARO (PF3D7_0414900), a rhoptry‐localized protein, shown to be palmitoylated on either or both of 2 cysteines within the first 20 amino acids of its N‐terminus (Cabrera *et al.,* 2012). It is important to note that little is currently known about DHHC‐PAT substrate specificity in any eukaryotic species, so some background palmitoylation of these targets could occur due to the presence of endogenous DHHC‐PATs in HEK293E cells. As a control, each target protein was therefore also expressed in the absence of a PfDHHC. If a target protein is more highly palmitoylated in the presence of the PfDHHC protein than in its absence, this would be a clear indication of its enzymatic activity. As an additional control for non‐specific purification of proteins after click chemistry and streptavidin affinity purification, co‐transfected HEK293E cells were also incubated with DMSO instead of 17‐ODYA.

### *Plasmodium falciparum* DHHC proteins can palmitoylate PfSec22

HEK293E cells were transiently transfected with each of the four PfDHHCs and the two target proteins, and their expression was determined by immunofluorescence. Antibodies against the C‐terminal FLAG tag present on the PfDHHCs and c‐Myc tag present on the targets confirmed protein expression for all transfected constructs, and showed that PfDHHC3 and 9 as well as PfSec22 localizes to the ER in HEK293E cells, while PfDHHC7 and PfARO localize to the plasma membrane (PM) (Figure S1). PfDHHC5 did not appear to localize to either the ER (Figure S1) or the PM (data not shown). However, PfDHHC5 contains ankyrin repeats in its N‐terminus, and other N‐terminal ankyrin repeat‐containing DHHC proteins, such as DHHC17 in humans (Yanai *et al.,* 2006) and Akr1 in yeast (Roth, 2002), have been localized to the Golgi; such a location for PfDHHC5 was not confirmed due to lack of appropriate antibodies, and because localization of the *P. falciparum* DHHCs in HEK293E cells would not necessarily affect their activity in this assay, which was the primary goal of this study.

**Figure 2:**
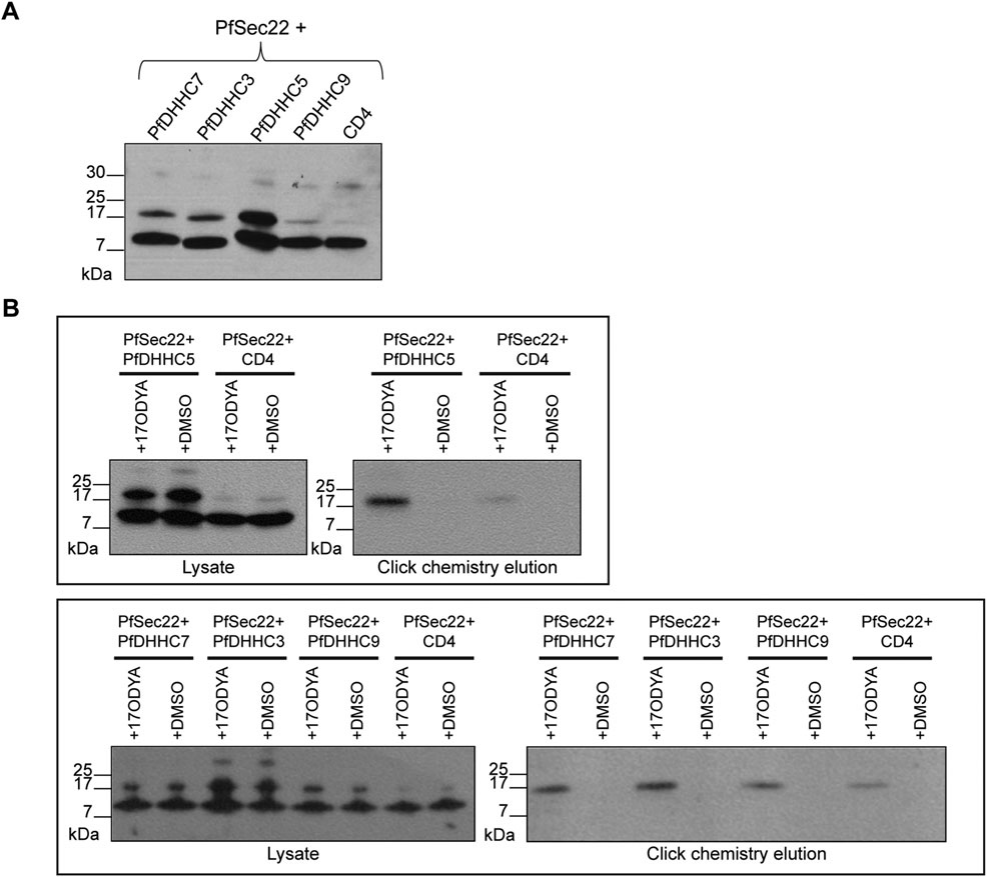
Palmitoyl‐transferase activity assay demonstrating the palmitoyl‐transferase activity of the PfDHHC proteins on PfSec22. A. Immunoprecipitation of PfSec22 co‐expressed with each PfDHHC protein. Human embryonic kidney 293E cells were co‐transfected with plasmids coding for the expression of c‐Myc‐tagged PfSec22, along with the indicated FLAG‐tagged PfDHHC proteins (PfDHHC3, 5, 7 and 9) or the control vector (CD4). Pfsec22 was immunoprecipitated from cell lysates using *α*‐c‐Myc antibody. The proteins were separated by SDS‐PAGE and visualized by immunoblot, using *α*‐c‐Myc antibody from a different species. B. PAT activity assay. Human embryonic kidney 293E cells were co‐transfected with plasmids expressing c‐Myc‐tagged PfSec22, along with the indicated FLAG‐tagged PfDHHC protein or the control vector (CD4). Cells were either treated with the metabolic label, 17‐octadecynoic acid (17‐ODYA) or mock‐treated with DMSO. Proteins were extracted and an aliquot of each lysate kept aside to confirm protein expression. The remaining lysates were put through click chemistry reactions to biotin‐azide, and 17‐ODYA‐labelled proteins were streptavidin affinity purified and eluted by boiling in SDS. Samples from the initial lysates and the click chemistry elutions were separated by SDS‐PAGE, and the presence of c‐Myc‐tagged PfSec22 in each of the samples was observed by immunoblot using antibodies against the c‐Myc tag.

To test for *P. falciparum* DHHC‐PAT activity, PfSec22 was expressed in HEK293E cells either in the presence of one of the PfDHHCs of interest (PfDHHC3, 5, 7 or 9) or with a CD4‐expressing control vector. PfSec22 was then immunoprecipitated from protein extracts and its expression confirmed by immunoblot using antibodies against the C‐terminal c‐Myc tag. Immunoblot revealed two clear PfSec22 bands: a stronger band at approximately 10 kDa and a weaker band at a higher molecular weight of approximately 17 kDa (Fig. 2A). Although protein palmitoylation does not always result in a shift in molecular weight, this has been observed for some proteins, such as the *P. falciparum* invasion motor complex protein, GAP45, which runs as a doublet where only the higher molecular weight band corresponds to palmitoylated GAP45 (Rees‐Channer *et al.,* 2006). This raises the possibility that the higher molecular weight band of PfSec22 corresponds to palmitoylated PfSec22.

To confirm that PfSec22 is getting palmitoylated, we performed the PAT activity assay (Fig. 1). After click chemistry and streptavidin affinity purification, immunoblot of the purified palmitoylated proteins with a c‐Myc antibody revealed a single band running at approximately 17 kDa (Fig. 2B). This band was present only in 17‐ODYA‐treated samples and not in DMSO‐treated samples, indicating that this was a palmitoylated form of PfSec22. The size of this band, and the absence of any other c‐Myc‐tagged species, establishes that the higher molecular weight band observed previously in the immunoprecipitates of PfSec22 is palmitoylated PfSec22, while the lower molecular weight band corresponds to non‐palmitoylated PfSec22 (Fig. 2A).

Palmitoylated PfSec22 was present when PfSec22 was expressed with all four PfDHHCs (Fig. 2B), and was also present at low levels when PfSec22 was expressed in the absence of a PfDHHC (Fig. 2B), most likely due to background palmitoylation by endogenous HEK293E DHHC‐PATs. Because there will be differences in transfection efficiencies, 17‐ODYA labelling and streptavidin affinity purification, these PAT activity assays are not robustly quantifiable. However, Fig. 2A shows that total PfSec22 expression levels were relatively similar in all transfections, and palmitoyl‐PfSec22 levels were clearly increased in PfDHHC‐expressing cells. This argues that co‐expression with each of the 4 PfDHHCs does indeed increase the level of PfSec22 palmitoylation, and is the first direct evidence of PfDHHC PAT activity.

### Plasmodium falciparum DHHC proteins can palmitoylate PfARO

**Figure 3:**
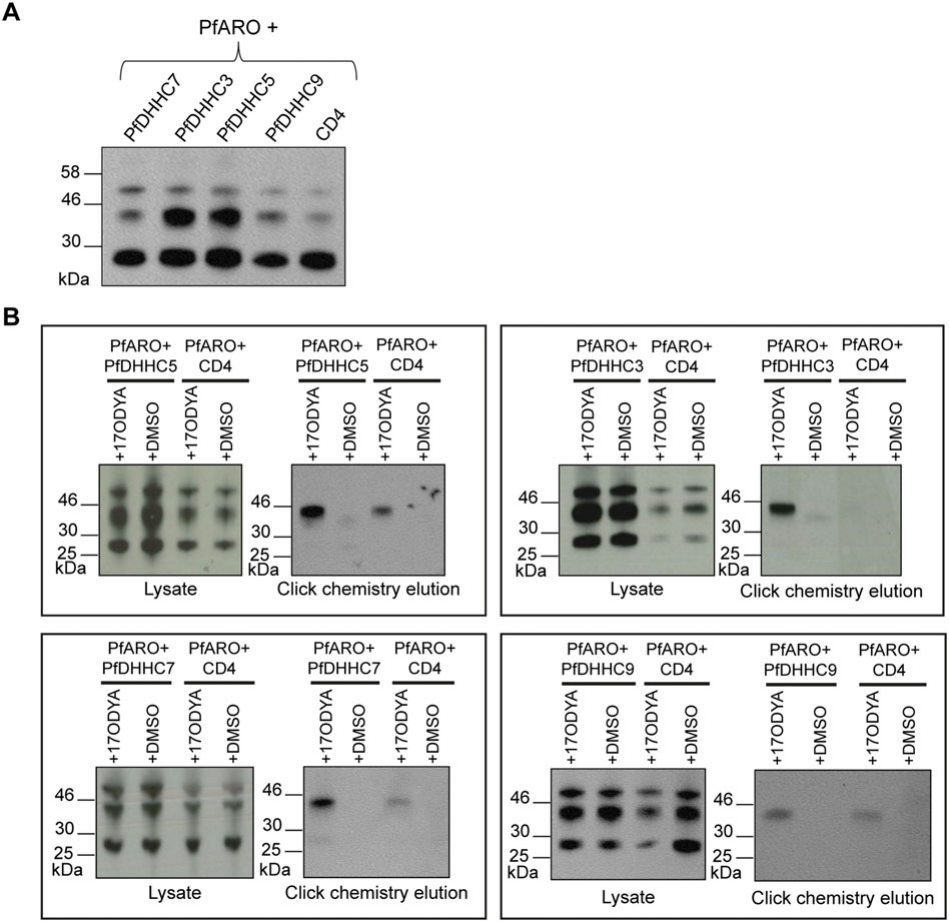
Palmitoyl‐transferase activity assay demonstrating the palmitoyl‐transferase activity of the PfDHHC proteins on PfARO. A. Immunoprecipitation of PfARO co‐expressed with each PfDHHC protein. Human embryonic kidney 293E cells were co‐transfected with plasmids coding for the expression of c‐Myc‐tagged PfARO, along with the indicated FLAG‐tagged PfDHHC proteins (PfDHHC3, 5, 7 and 9) or the control vector (CD4). PfARO was immunoprecipitated from cell lysates using *α*‐c‐Myc antibody. The proteins were separated by SDS‐PAGE and visualized by immunoblot, using *α*‐c‐Myc antibody from a different species. B. PAT activity assay. Human embryonic kidney 293E cells were co‐transfected with plasmids expressing c‐Myc‐tagged PfARO, along with the indicated FLAG‐tagged PfDHHC protein or the control vector, CD4. Cells were either treated with the metabolic label, 17‐octadecynoic acid or mock‐treated with DMSO. Proteins were extracted, and an aliquot of each lysate kept aside to confirm protein expression. The remaining lysates were put through click chemistry reactions to biotin‐azide, and 17‐octadecynoic acid‐labelled proteins were streptavidin affinity purified and eluted by boiling in SDS. Samples from the initial lysates and the click chemistry elutions were separated by SDS‐PAGE, and the presence of c‐Myc‐tagged PfARO in each of the samples was observed by immunoblot using antibodies against the c‐Myc tag.

PfARO was co‐expressed in HEK293E cells with each of the four PfDHHCs (PfDHHC3, 5, 7 and 9) and palmitoylation assayed as above. Immunoblot revealed two clear PfARO bands: a lower molecular weight band at approximately 28 kDa and a higher molecular weight band at approximately 45 kDa. Additionally, a faint third band was observed running above the 46 kDa marker (Fig. 3A). Immunoblot after 17‐ODYA labelling and palmitoylome purification identified a single band running at approximately 45 kDa, which was absent in DMSO‐treated samples (Fig. 3B). As with PfSec22, this higher mobility band appears to be the palmitoylated band of PfARO, while the lower molecular weight band at 28 kDa corresponds to the non‐palmitoylated version. The faint third band running above the 46 kDa marker was not observed in the click chemistry elutions, indicating that this band did not correspond to doubly‐ or multiply‐palmitoylated PfARO. This band could instead correspond to a version of PfARO, which was additionally modified by some other PTM, such as phosphorylation.

As with PfSec22, PfARO was subject to some background palmitoylation by endogenous HEK293E DHHC‐PATs (Fig. 3B). PfARO expression in 17‐ODYA click chemistry experiments was also varied (Fig. 3B), reflecting again the fact that these click chemistry‐based PAT assays are not robustly quantitative; however, in the immunoblot of immunoprecipitated PfARO (Fig. 3A), total protein expression in each transfection condition was clearly similar, while the intensity of the PfARO palmitoylated band was consistently greater when co‐expressed with PfDHHCs, and was the greatest in the presence of PfDHHC3 and 5 (Fig. 3A). This is direct evidence that PfARO is palmitoylated by each of the PfDHHCs, and perhaps more robustly by PfDHHC3 and 5, demonstrating again that all 4 PfDHHCs studied here are indeed PATs and can modify *P. falciparum* targets.

### PfARO is palmitoylated on multiple cysteines

**Figure 4:**
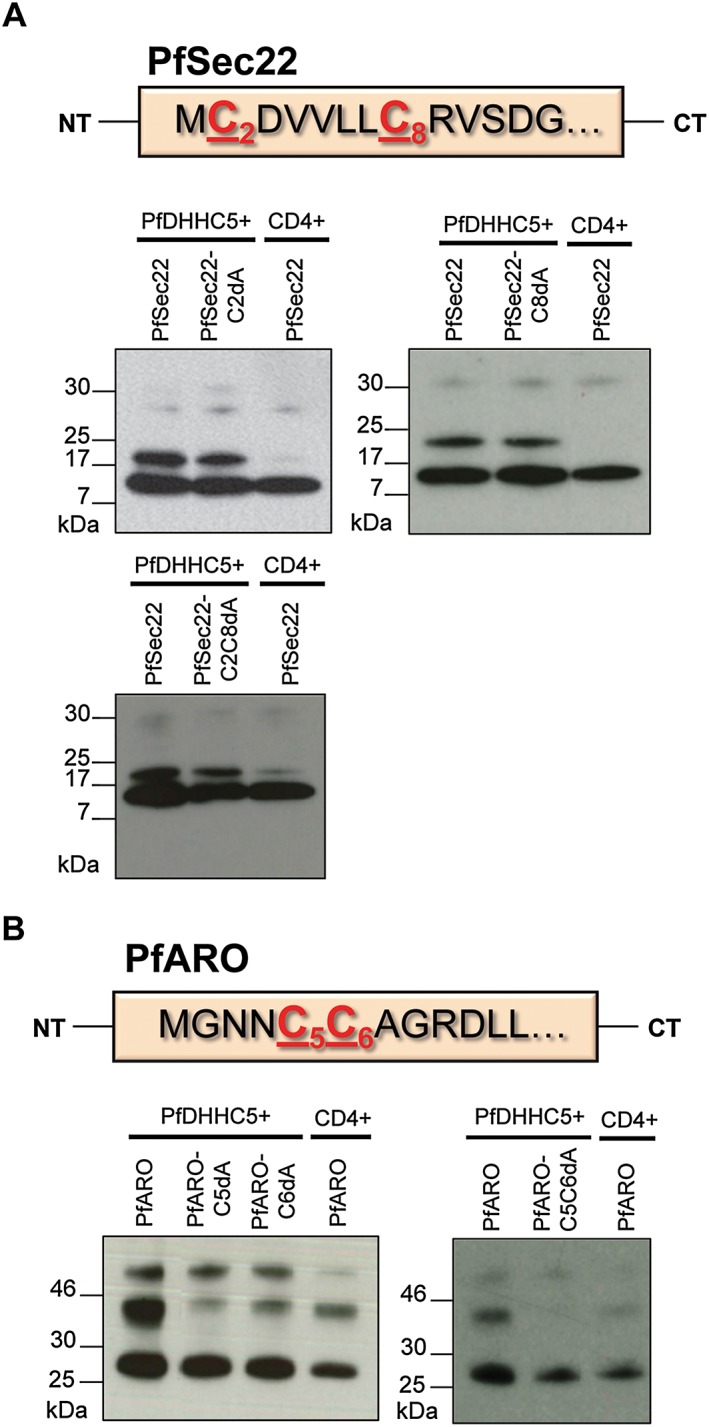
Immunoprecipitation of mutant PfSec22 and PfARO proteins using antibodies against the c‐Myc tag. Human embryonic kidney 293E cells were co‐transfected with plasmids coding for the expression of mutant c‐Myc‐tagged PfSec22 or PfARO (which had the predicted palmitoylated cysteine residues mutated to alanine residues), along with FLAG‐tagged PfDHHC5 or the control vector (CD4). The mutant PfSec22 and PfARO proteins were immunoprecipitated from cell lysates using *α*‐c‐Myc antibody. The proteins were separated by SDS‐PAGE and visualized by immunoblot, using *α*‐c‐Myc antibody from a different species. A. Immunoprecipitation of PfSec22 point mutants (PfSec22‐C2dA, PfSec22‐C8dA and PfSec22‐C2C8dA) along with wild‐type PfSec22. The amino acid sequence of the N‐terminal region of PfSec22 is also shown with the cysteine residues of interest highlighted in red. B. Immunoprecipitation of PfARO point mutants (PfARO‐C5dA, PfARO‐C6dA and PfARO‐C5C6dA) along with wild‐type PfARO. The amino acid sequence of the N‐terminal region of PfARO is also shown with the cysteine residues of interest highlighted in red.

The PAT activity assay above has established that PfSec22 and PfARO are both subject to palmitoylation by multiple PfDHHCs, indicating that these PATs have potentially broad substrate specificities. To establish whether a more stringent specificity exists at the target level, point mutations were introduced to eliminate putative palmitoylation sites of PfSec22 and PfARO. The mutated target proteins were all co‐expressed with PfDHHC5, which appears to robustly palmitoylate both targets. Palmitoylation sites are largely not predictable based on primary sequence, but preliminary experiments aimed at defining palmitoylation sites across the *P. falciparum* schizont proteome suggested that two cysteines in the N‐terminal domain of PfSec22 are palmitoylated (Cys2 and Cys8; Tay, Rayner & Choudhary, unpublished). However, introduction of point mutants in either or both of these cysteines (PfSec22‐C2dA, PfSec22‐C8dA and PfSec22‐C2C8dA) had no effect on PfSec22 palmitoylation in these assays (Fig. 4A), indicating that either PfSec22 is not palmitoylated at its N‐terminus by PfDHHC5, or that PfDHHC5 modifies multiple PfSec22 cysteines and mutation of just these two residues is insufficient to alter PfSec22 electrophoretic mobility. To distinguish between these two possibilities, a systematic analysis of all cysteines in PfSec22 will be required.

PfARO has previously been experimentally shown to be palmitoylated in *P. falciparum* parasites, and two cysteine residues in its N‐terminal region (Cys5 and Cys6) are predicted to be sites of palmitoylation (Cabrera *et al.,* 2012). Mutation of each site individually (PfARO‐C5dA, PfARO‐C6dA) reduced the intensity of the palmitoylated PfARO band relative to wild‐type PfARO, but did not eliminate it (Fig. 4B), indicating that perhaps PfDHHC5 requires both sites to be present in order to efficiently palmitoylate PfARO. To test this, we mutated both cysteines (PfARO‐C5C6dA), which abolished all PfARO palmitoylation by PfDHHC5 (Fig. 4B). Whether one or both residues is modified is not known, as the way in which palmitoylation affects the electrophoretic mobility of proteins is unpredictable, and does not usually correspond to the simple addition of the molecular weight of palmitate. Nevertheless, this caveat aside, this data suggests that Cys5 and Cys6 are the only sites involved in PfARO palmitoylation, at least by PfDHHC5, identifying a clear path for future *in vivo* functional analysis of PfARO palmitoylation.

### Expression and subcellular localization of DHHC proteins in *Plasmodium falciparum*

**Figure 5:**
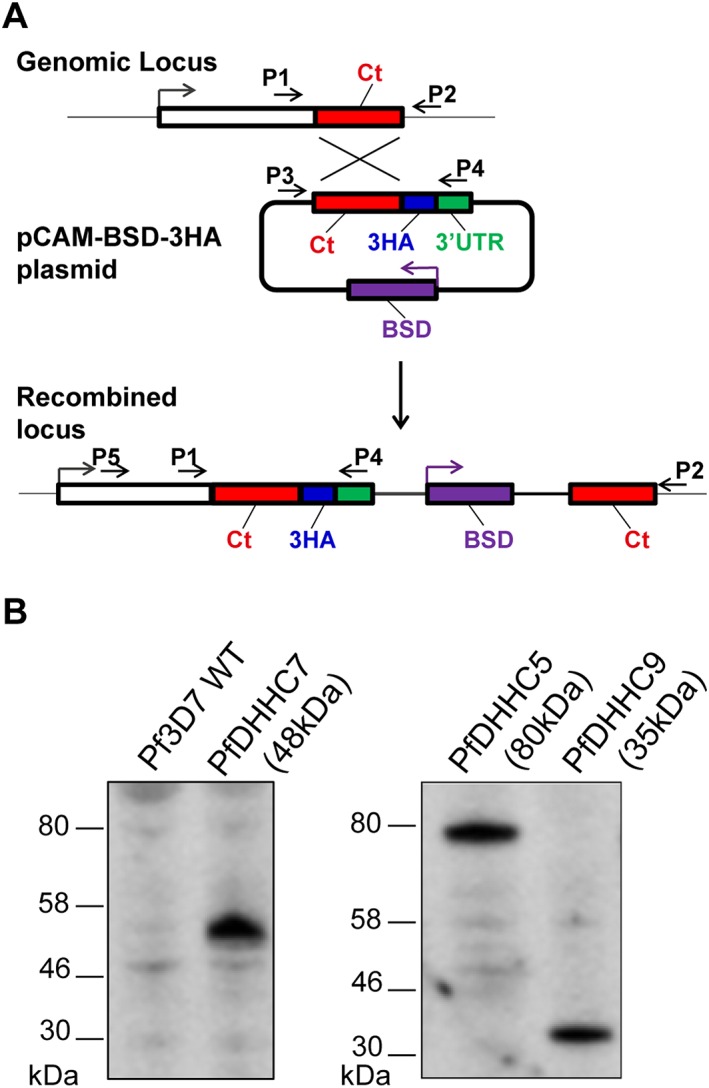
Generation of PfDHHC triple‐HA‐tagged transgenic lines. A. Scheme of the strategy used to C‐terminally‐tag the endogenous locus of the PfDHHC genes of interest with a 3‐HA epitope tag. The primer positions illustrated here indicate the primers used for genotyping of the PfDHHC 3‐HA‐tagged transgenic lines. Genotyping polymerase chain reaction of all 3‐HA‐tagged lines is shown in the supplementary material (Figure S2A). B. Immunoblot analysis was performed on total protein lysates from saponin‐lysed *Plasmodium falciparum* schizonts expressing triple‐HA‐tagged DHHC proteins. Membranes were probed with *α*‐HA antibodies, and the expected protein sizes are shown in brackets.

In the sections above, we successfully demonstrated that at least 4 of the PfDHHCs do indeed act as PATs. In order to determine the endogenous location of these schizont stage DHHC‐PATs within the parasite, each of the 4 DHHCs, as well as an additional schizont stage DHHC, PfDHHC8, were tagged by inserting a C‐terminal triple‐HA (3‐HA) epitope tag at the endogenous locus, allowing the tagged gene of interest to remain under the control of its native promoter (Fig. 5A). The insertion event for each of the PfDHHC genes was confirmed by genotyping polymerase chain reaction (PCR) (Figure S2A), and tagged protein expression determined by immunoblot of saponin‐lysed schizont pellets. Protein expression was detected by immunoblot for PfDHHC5, 7 and 9, each of which produced single bands that migrated at the expected size of the predicted translation product (Fig. 5B). Expression of PfDHHC3 and 8 was not detectable, perhaps due to low endogenous expression levels for these particular PfDHHCs, or due to the technical difficulty of extracting and solubilizing these multi‐TM‐domain membrane proteins. PfDHHC3, 5, 7 and 9 were all detectable in the parasite schizont stages by immunofluorescence, suggesting that in the case of PfDHHC3, the inability to detect the tagged protein by immunoblot is indeed likely due to difficulties in protein extraction. Expression of PfDHHC8‐3HA was not detectable even by immunofluorescence assay, suggesting that the expression of this PfDHHC may be at a level that is too low to be detected by tagging at the endogenous locus.

**Figure 6:**
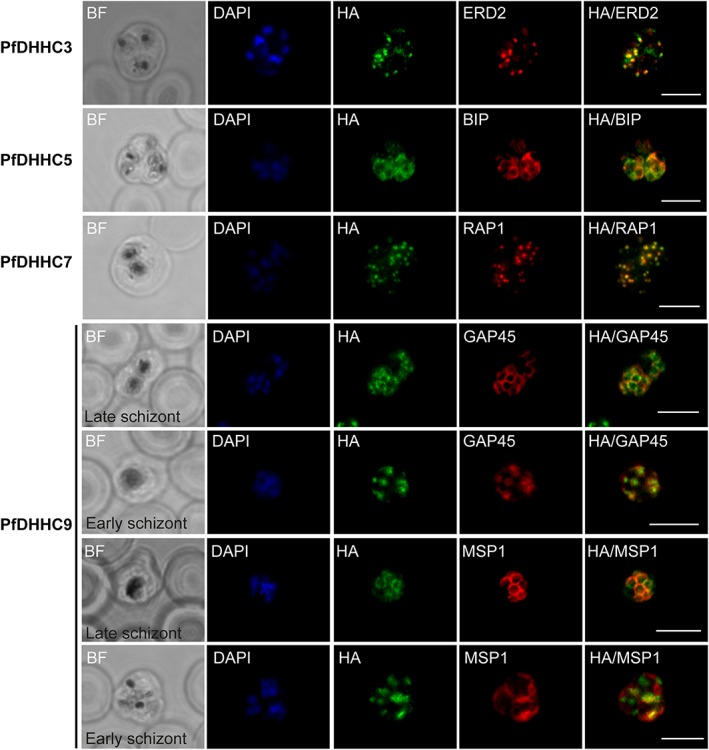
Expression and localization of PfDHHC proteins in *Plasmodium falciparum* schizonts. Triple‐HA‐tagged PfDHHC proteins were localized by immunofluorescence using antibodies against the 3‐HA tag (green). Immunofluorescence staining of each of the tagged PfDHHC proteins was compared against that of the following known localization markers (red): ERD2 (Golgi marker), BIP (endoplasmic reticulum marker), RAP1 (rhoptry marker), GAP45 (inner membrane complex marker) and MSP1 (plasma membrane marker). Nuclear staining by DAPI is shown in blue. For the staining of PfDHHC9 with GAP45 and MSP1, a late schizont, as well as an early schizont, is shown in order to differentiate between inner membrane complex and plasma membrane localization. Scale bar: 5 µm.

All four *P. falciparum* DHHC proteins were distributed to discrete intracellular locations in schizonts. PfDHHC3 immunofluorescence staining appeared punctate and co‐localized with ERD2 (Fig. 6), a protein that resides in the Golgi (Elmendorf and Haldar, 1993). PfDHHC5 staining co‐localized with BIP (Fig. 6), an ER protein (Van Dooren *et al.,* 2005). As previously reported, PfDHHC7 staining appeared punctate and was distinct from ERD2 staining but co‐localized with the rhoptry marker, RAP1 (Fig. 6) (Frénal *et al.,* 2013). Lastly, PfDHHC9 appeared to co‐localize with both MSP1, a marker for the PM (Holder *et al.,* 1992), and GAP45, a marker IMC (Langsley *et al.,* 2012), in late stage schizonts (Fig. 6). However, in the earlier stages of schizogony, PfDHHC9 co‐localized with GAP45, but did not co‐localize with MSP1 (Fig. 6). Although PM and IMC staining appears similar in mature schizonts, in earlier stages when the IMC is still developing, the PM and IMC can be distinguished (Kono *et al.,* 2012). Thus the co‐localization of PfDHHC9 with GAP45, the IMC marker, in both early and late stage schizonts suggests that PfDHHC9 is localized at the IMC, rather than the plasma membrane. It must be noted that the images shown in Fig. 6 are representative images and as such, provide an indication of co‐localization between the PfDHHCs and the relevant organelle markers. Further quantitative analysis would be required in order to further validate the co‐localization indicated here.

### PfDHHC5 and 9 can be disrupted in *Plasmodium falciparum*

**Figure 7:**
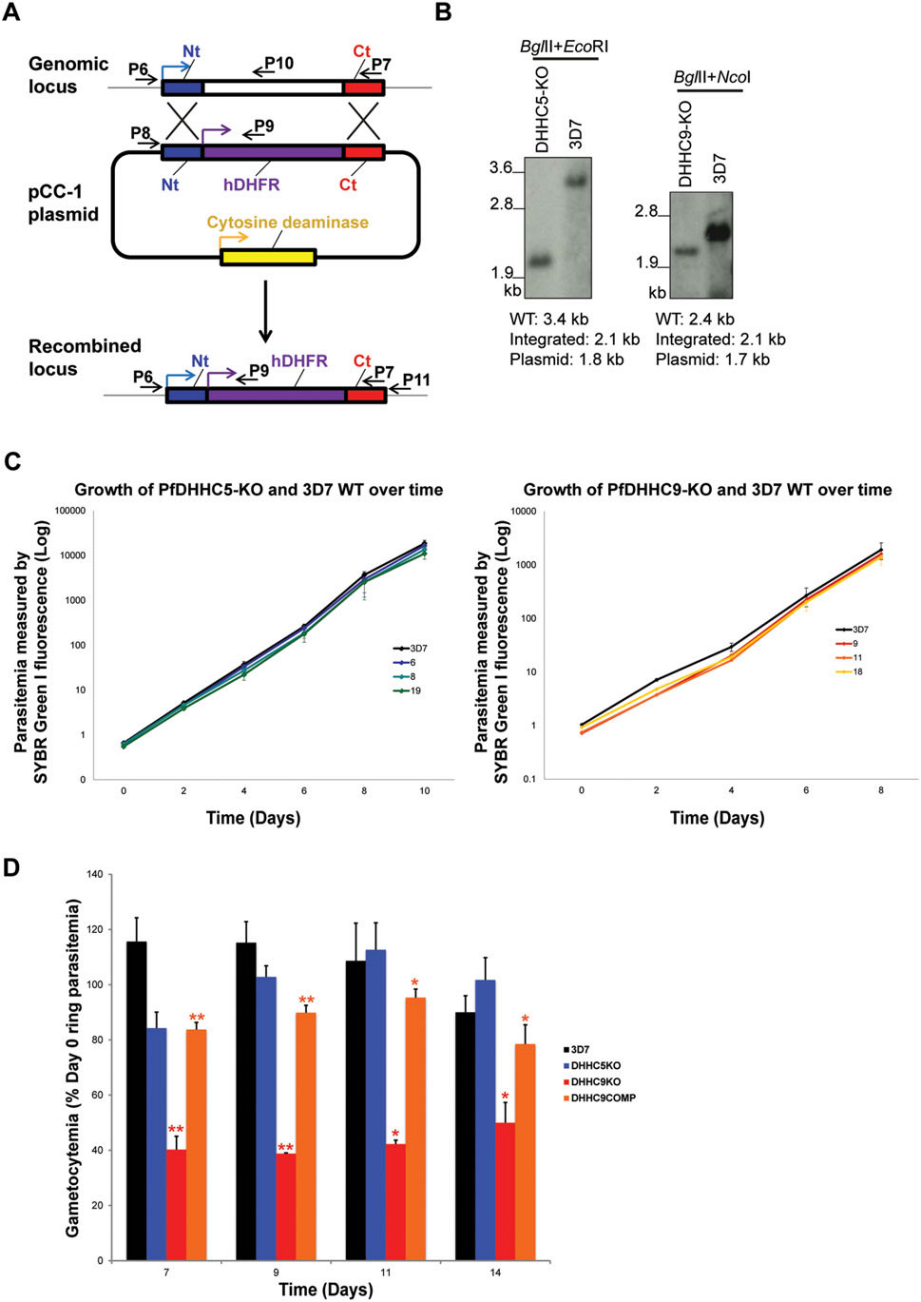
Generation of PfDHHC knock‐out transgenic lines in *Plasmodium falciparum*. A. Scheme of the strategy used to knock‐out the endogenous locus of the PfDHHC genes of interest. The primer positions illustrated here indicate the primers used for genotyping of the PfDHHC knock‐out transgenic lines. Genotyping PCR for PfDHHC5 and 9 knock‐out lines is shown in the supplementary material (Figure S2B). B. Southern blot confirming the disruption of PfDHHC5 and 9 knock‐out transgenic lines, using probes, which hybridized to the N‐terminal region of each gene. The restriction enzymes are used for digestion, and the expected sizes for the wild‐type, episomal and integrated locus are as indicated. (C) Growth assay comparing the growth of PfHHC5‐KO and PfDHHC9‐KO with wild‐type 3D7. Parasitemia of PfDHHC5‐KO clones (6, 8 and 19) (left) and PfDHHC9‐KO clones (9, 11 and 18) (right) along with wild‐type 3D7 measured every 2 days for a total of 8 or 10 days using flow cytometry. D. Gametocyte production in PfDHHC5KO, PfDHHC9KO and PfDHHC9COMP lines. Gametocytogenesis was induced in PfDHHC5 and 9 knock‐out parasites, as well as in PfDHHC9COMP parasites and wild‐type 3D7 by seeding cultures at 1% ring parasitemia (Day 0) and changing culture media daily for 14 days. The ring parasitemia on Day 2 was counted, and asexual growth was found to be similar across all lines (data not shown), as expected from the growth curves in (C). Percentage (%) gametocytemia was obtained by counting the number of gametocytes present in approximately 2000 erythrocytes on Day 7, 9, 11 and 14 after induction. The % gametocytemia was normalized to the 1% starting ring parasitemia on the day of induction (Day 0). The data are the result of three replicates ± standard error of the mean. 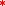 *P* ≤ 0.05, 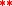 *P* ≤ 0.01 (unpaired t‐test, comparison between DHHC9KO and 3D7). 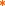 *P* ≤ 0.05, 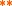 *P* ≤ 0.01 (unpaired t‐test, comparison between DHHC9KO and DHHC9COMP). Average gametocytemias along with the standard error of mean for each parasite line on each day is shown in Table S6.

Given that each DHHC protein is localized to a distinct membrane, we hypothesized that they might be less likely to overlap functionally. To test whether individual DHHC genes were essential for *P. falciparum* intraerythrocytic development, attempts were made to delete the majority of the coding sequence for each of the same 5 PfDHHCs (PfDHHC3, 5, 7, 8 and 9) using double‐cross‐over homologous recombination (Fig. 7A) (Maier *et al.,* 2006). Despite multiple attempts, integration of the knock‐out construct into the genome was detected for only 2 PfDHHCs: the ER‐localized PfDHHC5 and the IMC‐localized PfDHHC9. Genotyping by PCR of cloned PfDHHC5 and 9 knock‐out lines (PfDHHC5KO and PfDHHC9KO) indicated successful integration via double cross‐over recombination (Figure S2B), and Southern blot analysis confirmed disruption of the gene locus (Fig. 7B)*.* Although transfection was successful for the remaining PfDHHCs (PfDHHC3, 7 and 8), parasites with successful integration could not be obtained, despite multiple attempts. While absence of a knock‐out line is not definitive confirmation of essentiality, the same loci could be modified by integration of a 3‐HA tag, suggesting that these loci are genetically tractable. The results described here therefore suggest that, like in *P. berghei*, a subset of the *P. falciparum* DHHC proteins is not amenable to disruption in intraerythrocytic stages, although repeat analysis with conditional genetic systems will be required to confirm this.

Growth of the PfDHHC5 and 9KO lines was indistinguishable from that of wild‐type 3D7 over an 8 to 10 day period (Fig. 7C). Next, we investigated the effect of disrupting PfDHHC5 and 9 on gametocytogenesis. PfDHHC5KO was able to form gametocytes at a similar rate to wild‐type parasites (Fig. 7D), while when gametocytogenesis was induced in the PfDHHC9KO line, a significant reduction in the formation of gametocytes was observed. Although early and late stage gametocytes (Stages II to V) were observed in PfDHHC9KO parasites (Figure S3B), the number of gametocytes formed was significantly lower compared with wild‐type parasites (Fig. 7D), indicating that although mature gametocytes could be formed, this capability was significantly impaired, thus implying that PfDHHC9 may play a role in gametocytogenesis.

In order to ensure that this deficiency in gametocytogenesis did not arise spontaneously during transfection and culture of the modified line, the PfDHHC9KO line was complemented with an episomally maintained *P. falciparum* expression plasmid expressing PfDHHC9 (C‐terminally‐tagged with 3‐HA tag) (this complemented line is hereby referred to as PfDHHC9COMP). Expression of the exogenous PfDHHC9 protein was confirmed by immunoblot using antibodies against the 3‐HA tag (Figure S3A). The PfDHHC9COMP line was able to produce both early and mature gametocytes (Figure S3B) at a significantly higher rate compared with PfDHHC9KO and at a rate nearly but not quite equal to that of wild‐type parasites (Fig. 7D), which could be due to the fact that PfDHHC9 expression in the episomal vector is under the control of a heterologous promoter. The promoter used in the PfDHHC9COMP plasmid was the AMA1 promoter, which as indicated by the AMA1 transcriptional profile has lower expression in gametocytes compared with the endogenous PfDHHC9 promoter (López‐Barragán *et al.,* 2011) and has slightly lower expression in Stage‐V gametocytes compared with Stage II (López‐Barragán *et al.,* 2011). However, the increased drug selection concentration used here is expected to further boost PfDHHC9 expression by the exogenous promoter in this plasmid.

Nevertheless, the almost complete rescue of gametocytogenesis in the presence of PfDHHC9COMP indicates that it was the deletion of PfDHHC9 that caused the defect in the induction of gametocytogenesis rather than an off‐target concomitant change during transfection and culture. This indicates for the first time a role for palmitoylation in regulating gametocytogenesis, a key *P. falciparum* developmental transition. To test whether PfDHHC9 localized to the IMC in gametocytes as it does in asexual blood stages (Fig. 6), we performed immunofluorescence on gametocytes induced in the PfDHHC9‐3HA and PfDHHC9COMP lines. Both lines had similar staining, indicating that PfDHHC9COMP is expressed as expected, but in neither case did PfDHHC9 staining co‐localize with GAP45, an IMC marker (Figure S3C). Further investigation of the localization of PfDHHC9 in gametocytes will require the development of further organelle markers for gametocyte staining.

## Discussion

While the *P. falciparum* genome contains 12 genes coding for proteins that contain the conserved DHHC signature motif of palmitoyl‐transferases, the localization and function of most remain unknown, and in no case has it been confirmed that these homologues actually possess palmitoyl‐transferase activity. In this study, we determined that the *P. falciparum* DHHC proteins are indeed involved in catalysing protein palmitoylation by co‐expressing *P. falciparum* DHHCs and targets in a heterologous cell system and testing for palmitoyl‐transferase activity. All four *P. falciparum* DHHC proteins studied possessed PAT activity and were able to palmitoylate the same substrate protein, indicating that the function of these DHHC proteins may overlap, mirroring the functional overlap of some DHHC proteins in other eukaryotic systems (Roth *et al.,* 2006; Hicks *et al.,* 2011).

The PAT activity assay that was developed here utilizes transient transfection in HEK293E cells. This approach has the advantage of rapidity and adaptability, which was used here for the testing of multiple targets and PATs in different combinations to assess target specificity and for using site‐directed mutagenesis of targets to investigate site specificity. These are major advantages and allow studies that would simply not be feasible in *P. falciparum* parasites themselves. For example, the PAT activity assay revealed that both Cys5 and Cys6 of PfARO are palmitoylated, but no other cysteines are modified, a finding that will enable further functional follow‐up of the role of PfARO palmitoylation and emphasizing the fact that palmitoylation sites are not predictable based on primary sequence alone. However, like any enzyme assay, the PAT assay is not completely representative of the *in vivo* system and has disadvantages. One is that because it relies on transient transfection (to increase throughput), there is some variability between transfections, making it not strictly quantitative. Differences in band intensity can therefore strongly indicate enzyme or site specificity, as they do here, but not confirm it. An additional disadvantage is that the intracellular localizations of the *Plasmodium* proteins in HEK293E cells can be quite different compared with their endogenous localizations in *P. falciparum*; for example, PfDHHC7 and PfARO, which localize to the rhoptries in *P. falciparum* (Fig. 6)*,* are found to localize to the plasma membrane in HEK293E cells (Figure S1), presumably because of the lack of rhoptries in mammalian cells. Such differences in location could lead to false negative or false positive results and emphasize the need for multiple controls, as were employed here, to allow interpretation.

These caveats aside, in the PAT assay PfDHHC5 displayed stronger PAT activity towards PfSec22. This result and the fact that both proteins are also localized to the ER in *P. falciparum* may indicate that PfSec22 is in fact a natural substrate of PfDHHC5. More puzzling is the fact that PfDHHC7 was also able to palmitoylate PfSec22 in HEK293E cells. In *P. falciparum,* where PfDHHC7 is localized to the rhoptries, it is possible that PfDHHC7 never comes into contact with PfSec22 and thus may not naturally palmitoylate PfSec22. Alternatively, a low level of PfSec22 palmitoylation could be occurring as PfDHHC7 passes through the ER on the way to the rhoptries, as presumably is also occurring in the HEK293E system, where PfDHHC7 localized to the PM while PfSec22 remained in the ER (Figure S1B). Where palmitoylation occurs is not well understood for most targets, and could well occur at a location that is not the final destination for that protein. For example, PfARO was palmitoylated by both PfDHHC3 and PfDHHC5 in the PAT assay (located in the ER/Golgi in HEK293E cells, Figure S1A), despite the fact that the majority of PfARO was located in the PM in HEK293E cells (Figure S1). The acylation‐status and substrate/PAT interaction of PfARO may depend on its specific membrane association at any given time, as is the case for N‐Ras and H‐Ras in *Saccharomyces cerevisiae* (Rocks *et al.,* 2005). These results contribute to the hypothesis that substrate specificity may be regulated by the localization of the DHHC proteins in relation to the localization of the particular substrate protein.

We have also characterized five out of the 12 PfDHHC proteins in the parasite itself, focusing on those that are more highly transcribed in the schizont stages. Similar to a previous study of the repertoire of DHHC proteins in the rodent malaria‐causing species, *P. berghei* and the related Apicomplexan species, *T. gondii* (Frénal *et al.,* 2013), the DHHC proteins in *P. falciparum* localized to different membrane‐bound compartments within the parasite. These included the ER and Golgi, where the DHHC proteins of other eukaryotic organisms are commonly localized to (Lobo, 2002; Roth, 2002), as well as specialized, parasite‐specific organelles such as the rhoptries and the IMC. The localization of most of the PfDHHCs studied here was similar to that of their *P. berghei* homologues, although one PfDHHC (PfDHHC3) had a different localization compared with its *P. berghei* homologue (Frénal *et al.,* 2013). This indicates that even between these two related *Plasmodium* species, differences do exist between the localization of the DHHC orthologues, perhaps because there is one less DHHC protein in *P*. *berghei*. The localization of PfDHHC9 to the IMC in asexual stages was however in contrast to findings in another study (Wetzel *et al.,* 2015). These differences could perhaps be attributed to the fact that in that study, the tagged version of PfDHHC9 was over‐expressed episomally under a non‐native promoter.

The fact that so many different DHHC proteins exist in most eukaryotic organisms has always been puzzling, especially considering the fact that these proteins have been found to be relatively promiscuous in terms of substrate specificity (Roth *et al.,* 2006). While differential localization is one possible explanation, in the case of *Plasmodium* parasites, developmental stage‐specific functions could also increase the requirement for multiple PATs. This hypothesis is supported by our attempts to disrupt five schizont expressed *P. falciparum* PATs. Two PfDHHC proteins (PfDHHC5 and 9) could be successfully disrupted in the blood stages without any detrimental effect on blood stage growth, implying a functional redundancy for these two DHHC proteins, just as has been observed in yeast (Roth *et al.,* 2006). However, three PfDHHCs of interest here (PfDHHC3, 7 and 8) were unable to be disrupted. While this is not proof of essentiality, it mirrors findings in *P. berghei*, where not all DHHCs could be deleted in blood stages (Frénal *et al.,* 2013). This indicates that functional redundancy in *Plasmodium* DHHCs is not universal.

Although PfDHHC9 was successfully disrupted in the asexual blood stages, the loss of PfDHHC9 resulted in a reduction in the production of gametocytes in *P. falciparum*, perhaps because its substrate, or set of substrates, is only required or only present in gametocytes. This deficiency was almost completely rescued when the PfDHHC9 knock‐out line was complemented with exogenously expressed PfDHHC9, indicating that the deficiency in gametocytogenesis was the result of PfDHHC9 deletion and not off‐target genetic changes during culture. A function for PfDHHC9 in gametocytogenesis is supported by a previous study, which found that a *P. falciparum* line containing a piggyBac transposon insertion 1 kb upstream of PfDHHC9 had reduced gametocyte production (Ikadai *et al.,* 2013). Additionally, the localization of PfDHHC9 in gametocytes was found to be different from its localization in the blood stages. It could thus be hypothesized that the fact that PfDHHC9 is not essential in the blood stages but is required in gametocytes may be related to its different localizations in these two stages. This implies that life‐cycle stage‐specific expression and perhaps localization may play an important role in regulating the substrate specificity of *Plasmodium* DHHCs and highlight the need for further study of the DHHC proteins in stages of the parasite life cycle other than the blood stages. In the case of PfDHHC9, it was not within the scope of this investigation to determine whether the Stage‐V gametocytes that did manage to be produced by the PfDHHC9KO line were capable of gamete formation or transmission to the mosquito. Further characterization of the effect of PfDHHC9 loss in these sexual stages may yield valuable insight into the role of palmitoylation in this essential parasite developmental stage.

In summary, we have demonstrated the PAT activity of the *P. falciparum* DHHC protein family by developing an assay that can now be used to validate specific palmitoylation sites in target *Plasmodium* proteins. We have also shown that the PfDHHC‐PATs are distributed to different cellular sites and consist of proteins that are both essential and non‐essential for the blood stages, implying that localization and expression in specific life‐cycle stages may play a role in regulating PfDHHC substrate specificity. Given the attractiveness of stage‐specific targets for transmission blocking drugs, further study on the role played by protein palmitoylation in *Plasmodium* and, in these stages in particular, may both provide new insight into this key developmental step, as well as more rigorously assess their potential as targets for drug development.

## Experimental procedures

### Generation of human embryonic kidney 293E plasmid constructs

The HEK293E plasmid constructs were generated essentially as previously described (Durocher *et al.,* 2002; Bushell *et al.,* 2008). Briefly, all potential N‐linked glycosylation sites (N‐X‐S/T) were removed from the sequences of *P. falciparum* proteins of interest by replacing the serine/threonine residues with alanine residues. Sequence coding either for the FLAG tag or the c‐Myc tag was added to the C‐terminal region of the proteins of interest, followed by a STOP codon, and the entire protein sequence flanked by NotI (5'‐end) and AscI (3'‐end) restriction sites. The FLAG‐tagged or c‐Myc‐tagged protein sequences were codon‐optimized for expression in mammalian cells and introduced into pTT3‐based expression vectors using *GeneART* gene synthesis services (Life Technologies). For PfSec22 and PfARO point mutants, primers containing the desired cysteine to alanine point mutations (listed in Table S1) were used to PCR amplify the genes of interest using the *GeneART*‐synthesized expression plasmids as templates. The mutated genes of interest were then re‐introduced into the same expression plasmids using T_4_ DNA ligase (NEB).

### Human embryonic kidney 293E cell culture and transfection

Human embryonic kidney 293E (HEK293E) cells were maintained in Gibco® Freestyle™ 293 expression media (Life Technologies) supplemented with 1% heat‐inactivated FBS essentially as previously described (Bushell *et al.,* 2008), except that cells were grown without shaking in T75 tissue culture flasks (at 1 × 10^6^ cells/flask) with 25 ml of media, instead of in suspension. Transient transfection of HEK293E cells was performed using polyethylenimine (PEI) as previously described (Durocher *et al.,* 2002; Bushell *et al.,* 2008).

### Purification of palmitoylated proteins by metabolic labelling and click chemistry (palmitoyl‐transferase activity assay)

Transiently transfected HEK293E cells were treated with 25 µm of the palmitic acid analogue, 17‐ODYA (Cayman Chemical) or mock treated with an equal volume of DMSO, 24 h after transfection. The 17‐ODYA labelling was allowed to continue for 6 h at 37°C under standard culture conditions. Cell pellets were collected and lysed in 1% Triton X‐100/50 mm Tris‐Cl pH 7.4/150 mm NaCl/protease inhibitor cocktail (Roche) for 30 min at 37°C with shaking. The click chemistry reaction was then performed as previously described (Martin and Cravatt, 2009). Briefly, 2 mg of each protein sample was incubated with the following chemicals in PBS: 100 µm biotin‐azide (Life Technologies), 1 mm Tris(2‐carboxyethyl)phosphine (TCEP; Sigma‐Aldrich), 100 µm Tris[(1‐benzyl‐1*H*‐1,2,3‐triazol‐4‐yl)methyl]amine (TBTA, Sigma‐Aldrich) dissolved in DMSO/*tert‐*butanol (20%/80%), and 1 mm CuSO4 (Sigma‐Aldrich). The reaction was allowed to continue for 1.5 h at room temperature with shaking. Biotinylated proteins were affinity purified by incubating with a 50 µl bed volume of streptavidin‐agarose (Thermo Scientific) for 2 h at room temperature with rotation. Proteins were eluted by incubating with 100 µl of 2% SDS/50 mm Tris‐Cl pH 7.4/5 mm EDTA for 5 min at 95°C with shaking.

### Generation of *Plasmodium falciparum* plasmid constructs

For 3‐HA‐tagged plasmid constructs, an approximately 1000 bp fragment from the 3' end of the open reading frame of the gene of interest, excluding the STOP codon, was PCR amplified from the genomic DNA of *P. falciparum* strain 3D7 using the primers listed in Table S2. The PCR fragment was digested using the appropriate restriction enzymes (listed in Table S2) and then introduced into the pCAM‐BSD‐3HA vector (Abdi *et al.,* 2010) using T_4_ DNA ligase (NEB). Generation of the double‐cross‐over knock‐out plasmid constructs was performed essentially the same, except that approximately 600–800 bp of the N‐terminal and C‐terminal region of the gene of interest was PCR amplified (using primers listed in Table S2) and introduced one fragment at a time into the pCC1 knock‐out vector (Maier *et al.,* 2006). For the PfDHHC9COMP plasmid, the entire coding sequence of PfDHHC9, along with a 3‐HA tag at the C‐terminal end and flanked by KpnI and XhoI restriction enzyme sites, was made using *GeneART* gene synthesis services (Life Technologies) and introduced into the pARL‐ama1 vector (a kind gift from Dr. Jake Baum, Imperial College London) using the KpnI and XhoI sites. The hDHFR selection cassette of the pARL‐ama1 vector was removed and replaced with the Blasticidin‐S‐deaminase selection cassette.

### *Plasmodium falciparum* culture, transfection and genotyping of transgenic lines

*Plasmodium falciparum* strain 3D7 was cultured in RPMI‐based media containing O^+^ human erythrocytes at 5% haematocrit and 10% O^+^ heat‐inactivated serum or 0.5% AlbuMAX II (Life Technologies), according to established methods (Trager and Jensen, 1976). Transfection of ring‐stage parasites was performed as described (Fidock and Wellems, 1997). Positive drug selection was performed 1 day post‐transfection using 2.5 µg ml^−1^ Blasticidin‐S (Fisher Scientific) or 2.5 nm WR99210 and maintained until stable parasite growth was obtained. To select for parasites that had integrated the construct via homologous recombination, the parasites were maintained in the absence of drug for 3 weeks, after which drug pressure was reapplied until stable parasite growth was again achieved. For parasites transfected with the PfDHHC9COMP plasmid, once stable parasite growth was obtained, the concentration of Blasticidin‐S was increased to 5 µg ml in order to increase PfDHHC9 expression by the episomally maintained plasmid. For parasites transfected with the pCC1 knock‐out vector, negative selection was performed with 1 µm 5‐fluorocytosine (Sigma‐Aldrich) as previously described (Maier *et al.,* 2006). Genomic DNA was extracted from *in vitro* blood stage cultures infected with *P. falciparum* transgenic parasites using a QIAamp DNA Blood Mini Kit (QIAGEN) according to manufacturer's instructions. Genotyping by PCR was performed for each transgenic line using the primers listed in Table S3. Genotyping by Southern blot was performed essentially as described (Southern, 2006). Probes targeting the N‐terminal region of each gene of interest were produced by PCR amplification using genomic DNA and the primers listed in Table S4. Southern blot hybridization of probes was performed using the DIG‐High Prime DNA Labelling and Detection Starter Kit II (Roche) according to manufacturer's instructions.

### Immunodetection of 3HA‐tagged *Plasmodium falciparum* proteins and c‐Myc‐/FLAG‐tagged human embryonic kidney 293E proteins

For Western blot analysis, transgenic schizont‐stage parasites were extracted from *in vitro* blood‐stage cultures by saponin lysis. The saponin pellets were lysed in 4% SDS/50 mm Tris‐Cl pH 7.4/150 mm NaCl/5 mm EDTA, in an appropriate volume that would result in a concentration of 5 × 10^8^ parasites/ml, for 30 min at 37°C. The lysate was resuspended in reducing 2X Laemmli sample buffer and resolved by SDS‐PAGE. Proteins of interest were detected using the antibodies listed in Table S5. Transfected HEK293E cells were lysed and analysed the same way. For immunofluorescence assay, *in vitro* blood‐stage cultures infected with transgenic *P. falciparum* strains were fixed in 4% formaldehyde/0.01% gluteraldehyde/PBS for 1 h at room temperature. The fixed cells were permeablized with 0.1% Triton X‐100/PBS for 10 min at room temperature and then blocked with 3% BSA/PBS for 1 h at room temperature. All antibodies used were diluted in 1% BSA/PBS and are as listed in Table S5. Cells were mounted in Prolong Anti‐fade mounting reagent (Life Technologies). For HEK293E cells, immunofluorescence assay was performed essentially the same, except that the cells were grown on poly‐l‐lysine coverslips (BD Biosciences) in 12‐well plates and all steps of the assay were performed on the coverslips. Confocal images were acquired using a Zeiss LSM 510 Laser scanning confocal microscope. For the immunoprecipitation of tagged proteins, cell pellets were lysed in immunoprecipitation buffer [1% Triton X‐100/50 mm Tris‐Cl pH 7.4/150 mm NaCl/5 mm EDTA/protease inhibitor cocktail (Roche)] for 30 min at 37°C with shaking. The lysates were pre‐cleared with 15 µl Protein G‐sepharose® (Sigma Aldrich) for 1 h at 4°C with rotation, followed by incubation with 2 µg of the appropriate antibody overnight at 4°C with rotation. The samples were then incubated with 30 µl Protein G‐sepharose for 2 h at 4°C with rotation and the immunoprecipitated proteins eluted from the resin by incubation with 60 µl 2%SDS/50 mm Tris‐Cl pH 7.4/5 mm EDTA for 5 min at 95°C with shaking.

### *Plasmodium falciparum* growth assay

*Plasmodium falciparum* blood‐stage culture was diluted to produce a suspension at 2% haematocrit and 1% parasitemia. A 10 µl aliquot of the parasite suspension was fixed in 2% formaldehyde/0.2% gluteraldehyde/PBS for 45 min at 4°C. The fixed cells (labelled ‘Day 0’) were kept in 50 µl of PBS until further use. The remaining parasite suspension (2% haematocrit and 1% parasitemia) was added into a 96‐well round‐bottomed plate at 100 µl volume per well. After 2 days incubation under *P. falciparum* standard culture conditions, a 10 µl of aliquot was again removed from the plate and fixed as described earlier (labelled ‘Day 2’). The cells in the plate were then diluted 1:5 or 1:10 with fresh red blood cells (2% haematocrit) and incubated for another 2 days under standard culture conditions. This was repeated every 2 days until ‘Day 8’ or ‘10’ cells were collected and fixed. The fixed cells were permeabilized in 0.3% Triton X‐100/PBS for 10 min at room temperature followed by incubation with 0.5 mg ml^−1^ ribonuclease A (MP Biomedicals) for 45 min at 37°C. Finally, the cells were stained with SYBR Green I (Life Technologies)/PBS, at a concentration of 1:5000, for 45 min at 37°C, followed by acquisition on a flow cytometer as previously described (Theron *et al.,* 2010). Parasitemia was determined by SYBR Green I fluorescence as measured by the flow cytometer. All experiments were carried out in triplicate, and the data were presented as mean ± standard deviation.

### Plasmodium falciparum gametocyte production

Gametocyte induction was performed as previously described (Lamour *et al.,* 2014). Briefly, each *P. falciparum* line was seeded at a starting parasitemia of 1% rings and at 4% haematocrit in 10 ml culture media (media as described earlier), without drugs (Day 0). The culture media was replaced daily for a total of 14 days. Ring parasitemia was checked on Day 2 in order to ensure that asexual growth was as expected for all parasite lines. Smears were taken on Days 7, 9, 11 and 14, and the number of gametocytes observed was counted. On Day 7, mostly Stage‐II gametocytes were observed, while on Day 9, mostly Stage‐III gametocytes were observed. On Day 11, mostly Stage‐IV gametocytes were observed, and on Day 14, mostly Stage‐V gametocytes were observed.

## Supporting information


**Fig. S1.** Localization of codon‐optimized *P. falciparum* proteins in HEK293 cells. (A) Codon‐optimized *P. falciparum* DHHC proteins (PfDHHC3, 5, 7 and 9) were expressed in HEK293E cells and expression of the proteins determined by immunofluorescence assay using antibodies against the FLAG tag. Localization of the PfDHHC proteins (green) was determined by comparing the immunofluorescence signal to that of the following known localization markers (red): calnexin (endoplasmic reticulum marker) and cadherin (plasma membrane marker). Nuclear staining by DAPI is shown in blue. (B) Codon‐optimized *P. falciparum* proteins PfARO and Pfsec22 were expressed in HEK293E cells. Expression was determined by immunofluorescence assay using antibodies against the c‐Myc tag. Both ARO and PfSec22 (green) were localized by immunofluorescence against the following mammalian localization markers (red): calnexin (endolasmic reticulum marker) and cadherin (plasma membrane marker). Nuclear staining by DAPI is shown in blue. Scale bar: 10 µm.
**Fig. S2.** Generation of PfDHHC triple‐HA‐tagged and knock‐out transgenic lines. (A) Genotyping of the PfDHHC tagged transgenic lines by genomic PCR analysis. Primer pairs used for the amplification of sequences specific to the wild‐type locus, episome and integrated construct are as listed in the table on the right, along with the expected sizes of the fragments. (B) Genotyping of the PfDHHC5 and 9 knock‐out lines by genomic PCR analysis. Primer pairs used for the amplification of sequences specific to the wild‐type locus, episome and double‐cross‐over integrated construct (DXO) are as listed in the table on the left, along with the expected sizes of the fragments.
**Fig. S3.** Expression and localization of exogenously expressed triple‐HA‐tagged PfDHHC9 in the PfDHHC9 knock‐out line (PfDHHC9COMP). The PfDHHC9 knock‐out line was complemented with exogenously expressed triple‐HA‐tagged PfDHHC9. (A) Immunoblot analysis was performed on total protein lysate from saponin‐lysed PfDHHC9COMP schizonts. The membrane was probed with α‐HA antibodies and the expected size is shown in brackets. (B) Representative images of Giemsa stained gametocytes on Day 7 (Stage II), Day 9 (Stage III), Day 11 (Stage IV) and Day 14 (Stage V) induced in the DHHC5KO, DHHC9KO and DHHC9COMP parasite lines, as well as in 3D7 parasites. Scale bar: 5 µm. (C) Expression of 3‐HA‐tagged PfDHHC9 in gametocytes induced in both the PfDHHC9‐3HA line and the PfDHHC9COMP line was determined by immunofluorescence assay using antibodies against the 3‐HA tag (green). PfDHHC9 immunofluorescence staining in both lines was compared against that of the inner membrane complex marker, GAP45 (red). Nuclear staining by DAPI is shown in blue. Scale bar: 5 µm.
**Table S1.** Primers used for the generation of point mutations in the PfARO and PfSec22 HEK293E expression plasmid constructs. The codon targeted for the point mutation is underlined and highlighted in red. The restriction enzyme sites are highlighted in red and indicated along with the vector used in the columns on the right.
**Table S2.** Primers used for the generation of *P. falciparum* triple‐HA‐tagged and knock‐out plasmid constructs. The restriction enzyme sites are highlighted in red and indicated along with the vector used in the columns on the right.
**Table S3.** Primers used for the genotyping of *P. falciparum* triple‐HA‐tagged lines and knockout transgenic lines.
**Table S4.** Primers used for the production of Southern blot hybridization probes targeting the N‐terminal region of each gene of interest.
**Table S5.** All primary and secondary antibodies used in this work along with their appropriate working dilutions.
**Table S6.** Average gametocytemia in PfDHHC5KO, PfDHHC9KO, PfDHHC9COMP and 3D7 parasite lines. Average gametocytemia of each parasite line is shown normalized to the starting ring parasitemia at Day 0 (1% rings) along with the standard error of mean (SEM).

Supporting info item

Supporting info item

Supporting info item

Supporting info item

Supporting info item

Supporting info item

Supporting info item

Supporting info item

Supporting info item
