# Supplementary figures and images for: Study of Plasmodium falciparum DHHC palmitoyl transferases identifies a role for PfDHHC9 in gametocytogenesis
Source: Cell Microbiol. 2016 May 3;18(11):1596–610. doi: 10.1111/cmi.12599 (PMC5091645; doi:10.1111/cmi.12599)

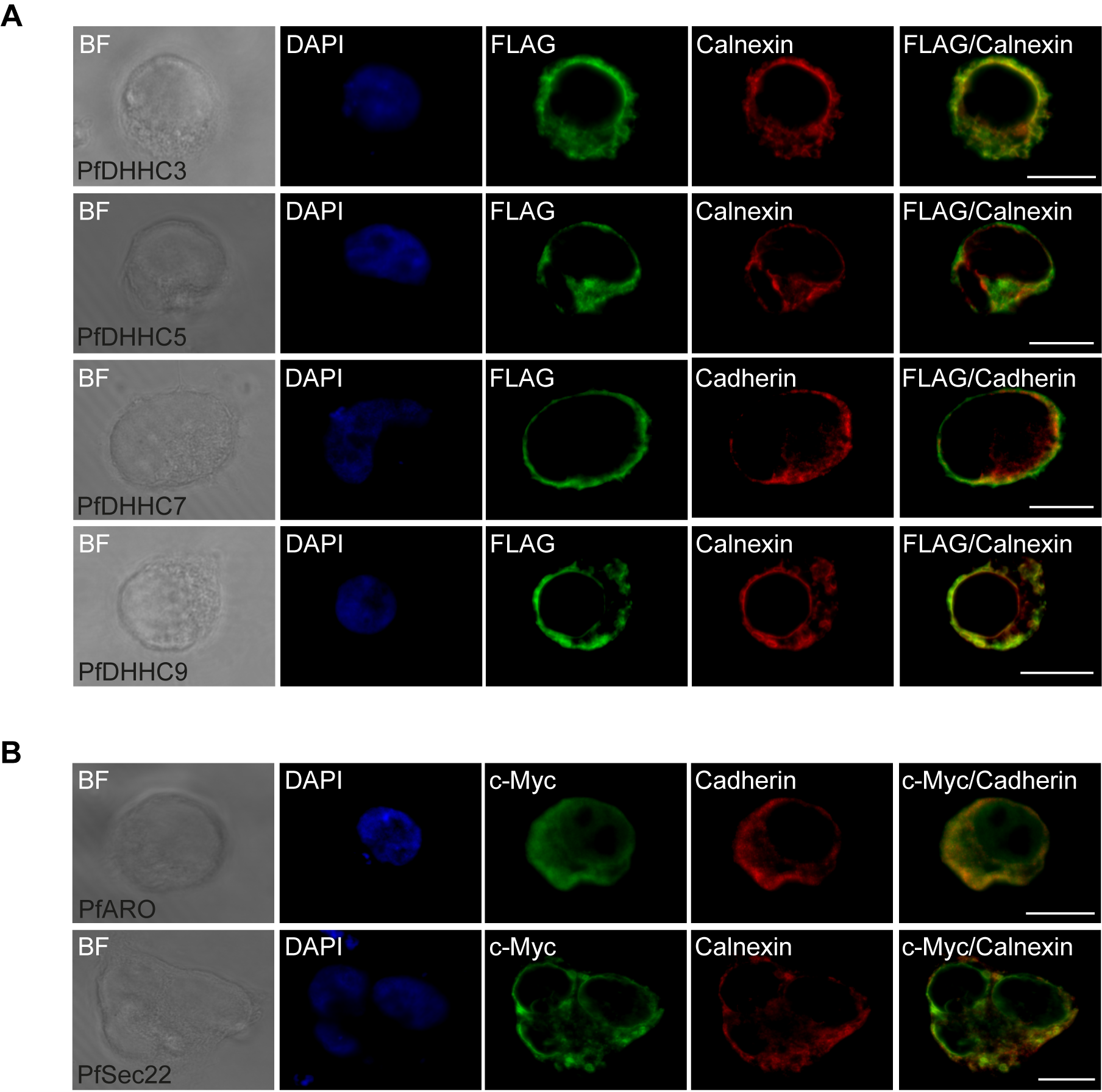

Supplement: Supplementary file 1 — Supporting info item [file CMI-18-1596-s001.tif]

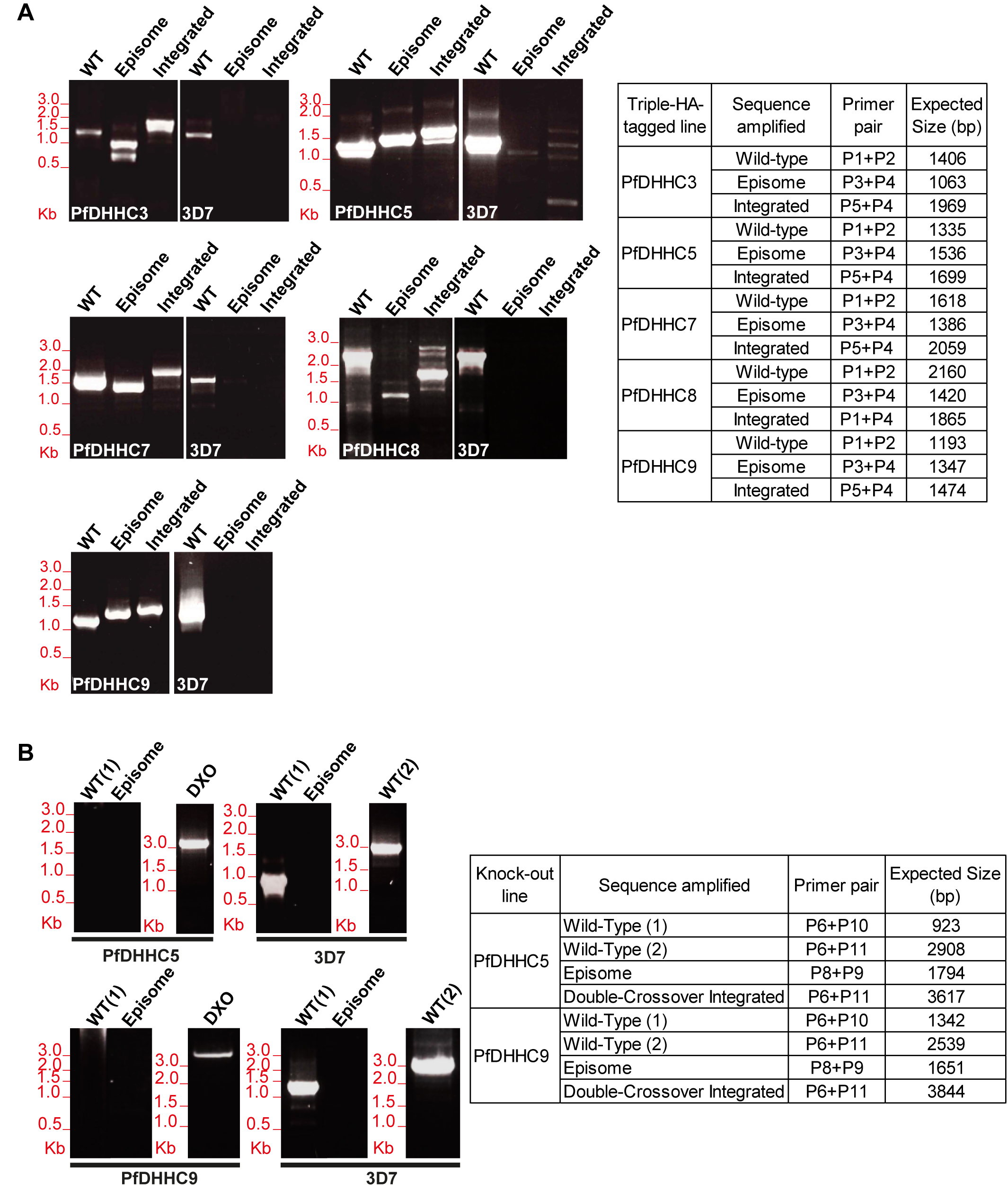

Supplement: Supplementary file 2 — Supporting info item [file CMI-18-1596-s002.tif]

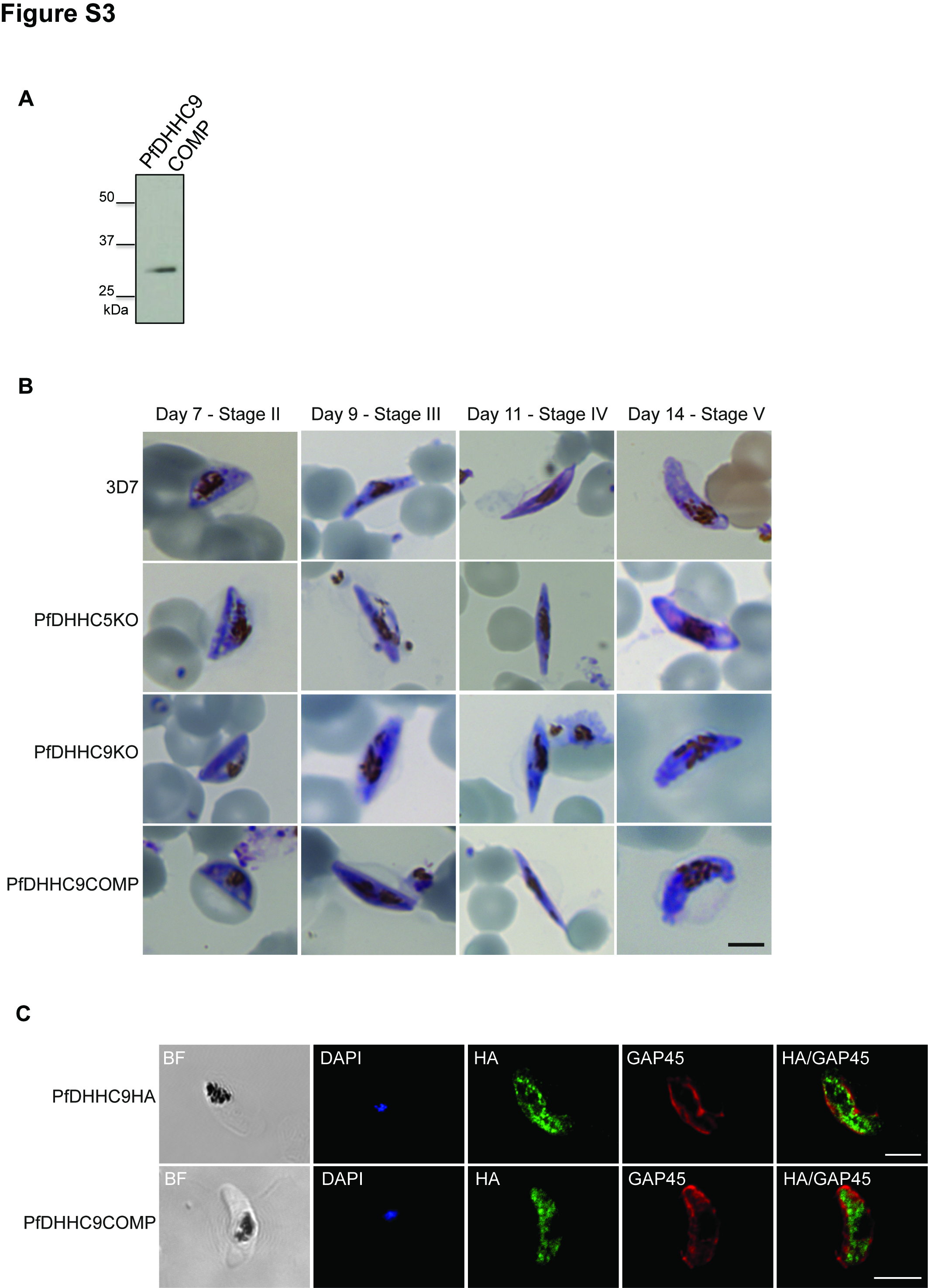

Supplement: Supplementary file 3 — Supporting info item [file CMI-18-1596-s003.tif]

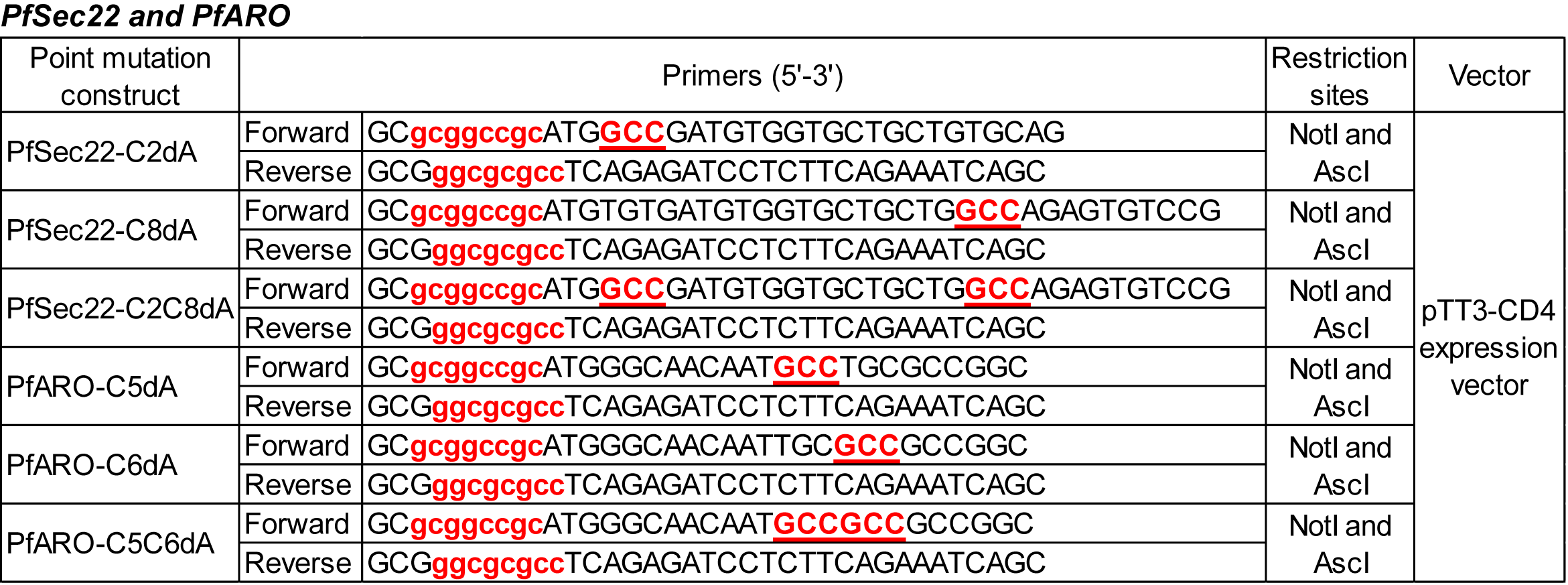

Supplement: Supplementary file 4 — Supporting info item [file CMI-18-1596-s004.tif]

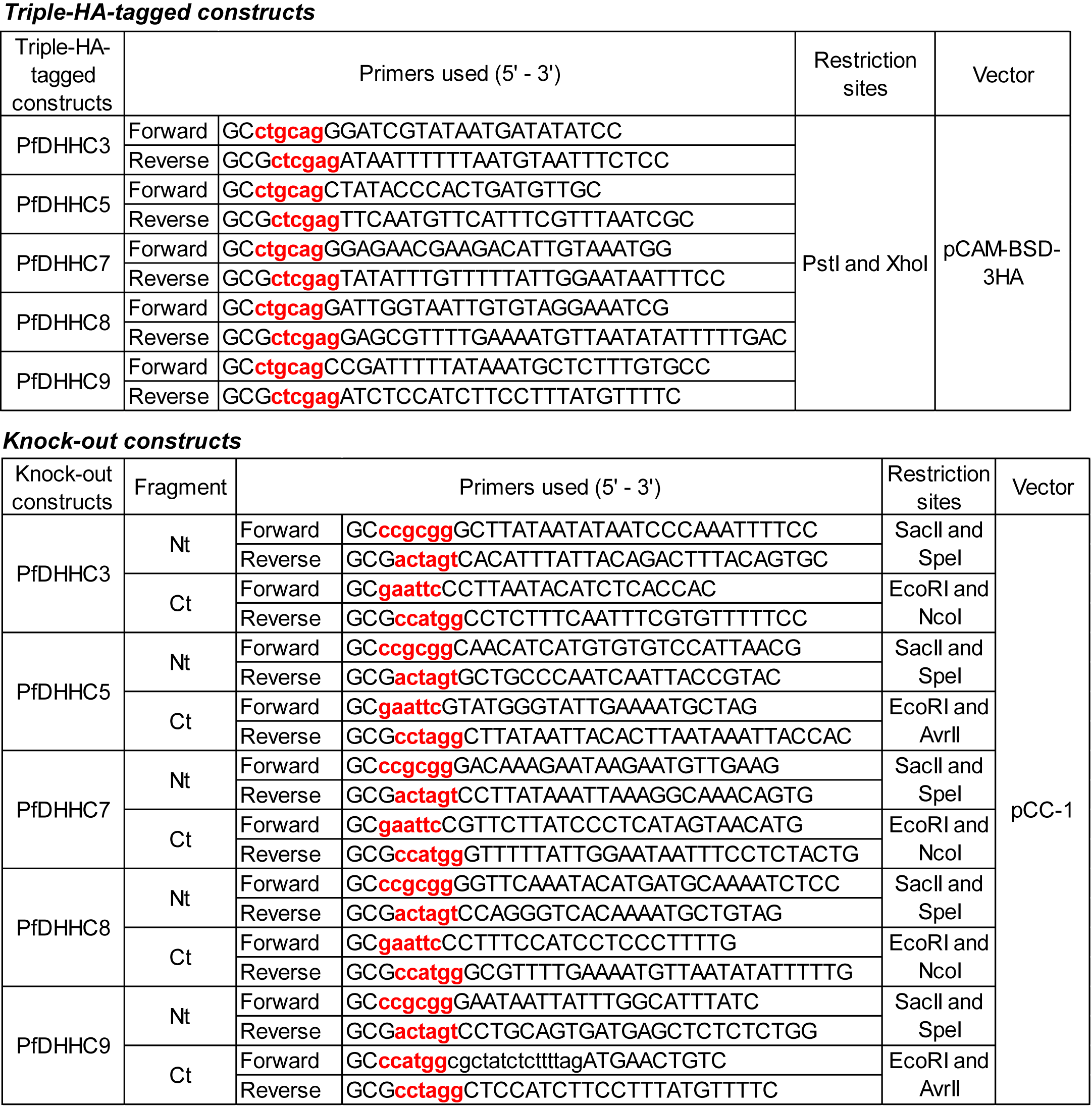

Supplement: Supplementary file 5 — Supporting info item [file CMI-18-1596-s005.tif]

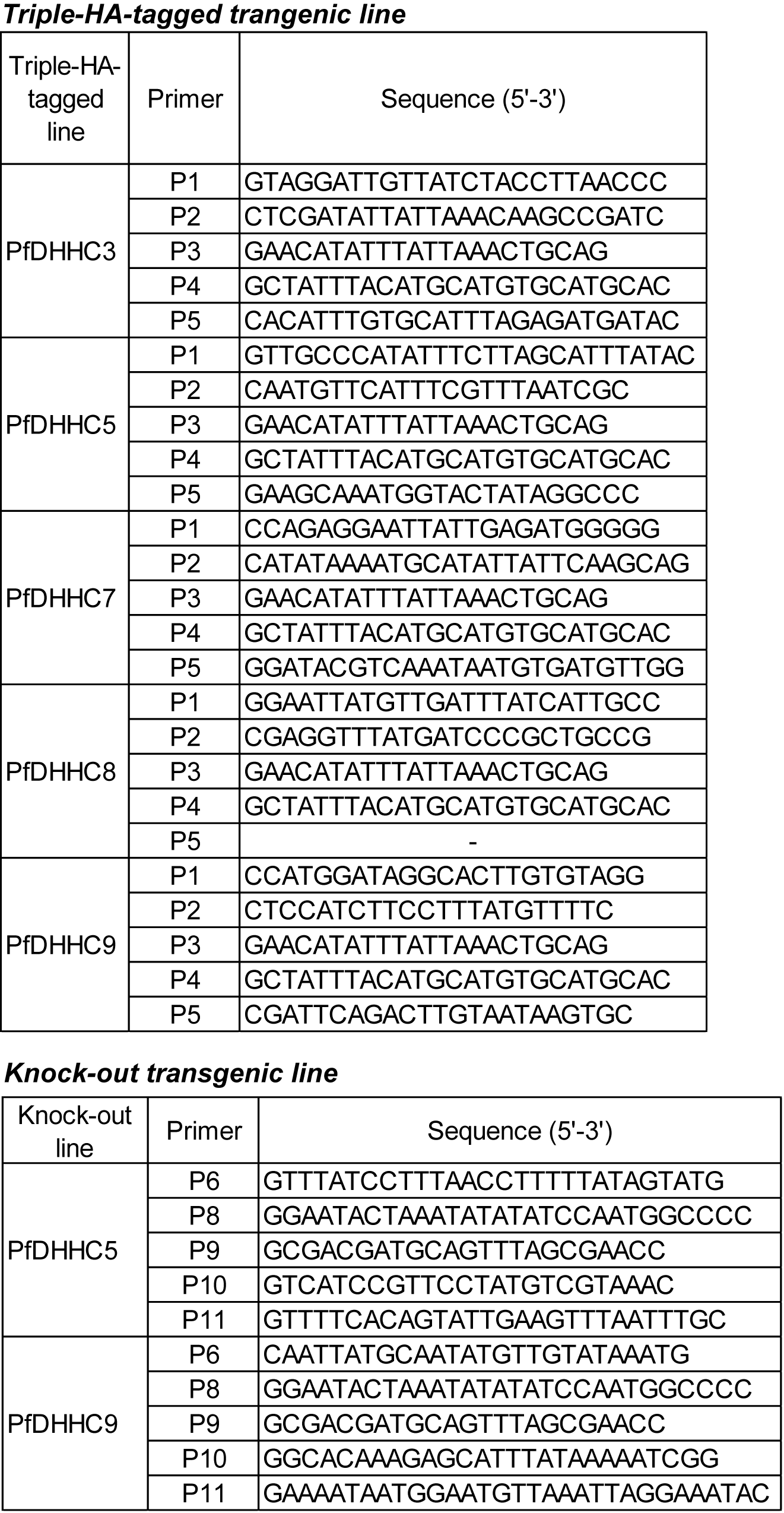

Supplement: Supplementary file 6 — Supporting info item [file CMI-18-1596-s006.tif]

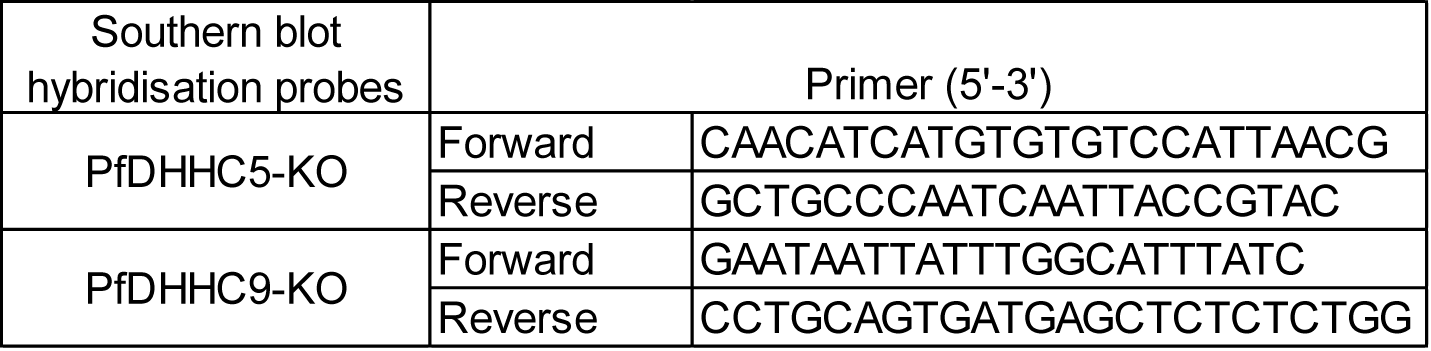

Supplement: Supplementary file 7 — Supporting info item [file CMI-18-1596-s007.tif]

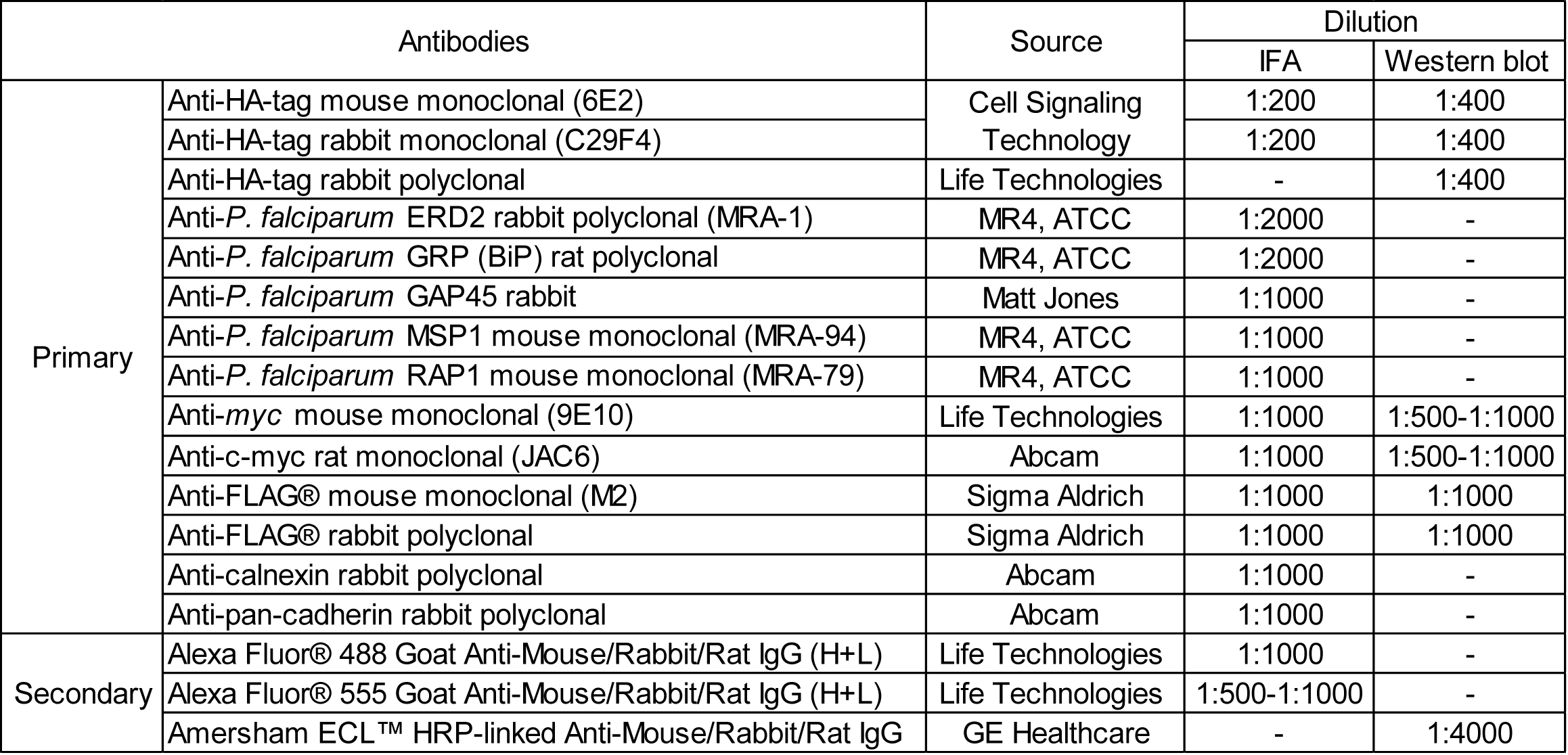

Supplement: Supplementary file 8 — Supporting info item [file CMI-18-1596-s008.tif]

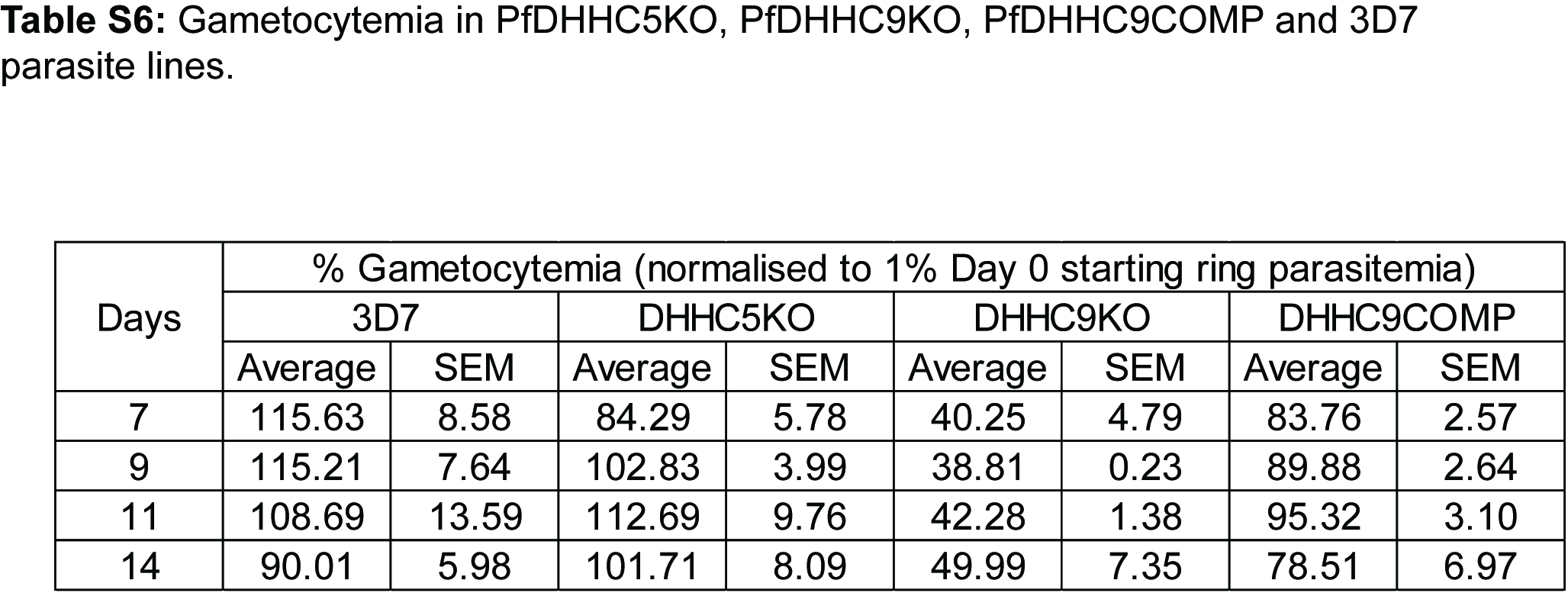

Supplement: Supplementary file 9 — Supporting info item [file CMI-18-1596-s009.tif]
